# A surface plasmon resonance-based method for monitoring interactions between G protein-coupled receptors and interacting proteins

**DOI:** 10.14440/jbm.2016.97

**Published:** 2016-01-28

**Authors:** Nikolas Stroth

**Affiliations:** Center for Molecular Medicine, Karolinska Institute and University Hospital, Stockholm, Sweden

**Keywords:** G protein-coupled receptor, protein-protein interaction, surface plasmon resonance, S100B, 5-HT_7_

## Abstract

The present protocol describes a method by which interactions between G protein-coupled receptors (GPCR) and intracellular proteins can be monitored in real-time and without the use of exogenous labels. The method is based on surface plasmon resonance (SPR) and uses synthetic peptides as mimics of intracellular GPCR domains. These peptides are covalently immobilized onto sensor chips and brought into contact with putative interacting proteins in the flow cells of the SPR instrument. The method allows flexible experimental designs, rapid testing of hypotheses and quantitative analysis of interactions. Relative to other established methods, it provides both an alternative and a complementary approach with several key advantages. The present protocol describes the method step-by-step, using the interaction between the serotonin 5-HT_7_ receptor and the calcium-binding protein S100B as an example.

## INTRODUCTION

G protein-coupled receptors (GPCR) are the targets for more than 25% of currently available drugs [1] and represent 15% of the “druggable genome” [2]. Advances in our understanding of GPCR structure and function will continue to elucidate their roles in basic biology and inform GPCR-directed drug discovery projects [3, 4]. Experimental methods based on surface plasmon resonance (SPR) have been used to study protein-protein interactions of GPCR and their associated signaling machinery [5-7]. One main advantage of SPR-based methods is their ability to provide real-time, label-free measurements of molecular interactions from which information concerning affinity, kinetics, concentration etc. can be derived. The principles and many applications of SPR have been extensively reviewed [8] and will not be recounted here in detail. Briefly, SPR-based methods require that one interacting molecule (commonly termed “ligand”) is immobilized on a solid surface (sensor chip) while the other molecule (“analyte”) is brought into contact via the flow cells of an SPR instrument. Binding of the analyte to the ligand changes the mass associated with the sensor chip surface, thus altering the refractive index of the surface. The refractive index is continuously monitored by the instrument’s detector, translated into “response units” within the instrument and displayed by an attached computer. In short, SPR instruments function as “refractometric sensing devices” [8]. In the present protocol, a method is described that uses one such device (Biacore 3000) to study interactions between a GPCR and an intracellular interacting protein. In principle, it is similar to previously published methods in which synthetic peptides were used to mimic domains of a GPCR in lieu of the entire protein [9-11]. It is possible to immobilize full-length GPCR on sensor chips (see for example [12-14]), but the speed and simplicity of the present method make it preferable in various circumstances and especially useful for rapid hypothesis testing.

## MATERIALS

The present protocol is based on a Biacore 3000 instrument and uses several ready-made buffers and solutions sold by the instrument provider (GE Healthcare Europe, Uppsala, Sweden). Since the recipes are openly available, these buffers and solutions (as well as customized modifications) can easily be prepared in the laboratory.

### Equipment and software

• Biacore 3000 SPR instrument (includes PC and instrument control software; Cat. # BR110045; GE Healthcare)
• Vacuum pump for filtration and degassing of buffers and solutions
• Milli-Q Advantage A10 Ultrapure Water Purification System (Merck Chemicals and Life Science AB, Solna, Sweden)
• BIAevaluation Software 4.1.1 (Cat. # BR100216; GE Healthcare)
• GraphPad Prism 5.04 (GraphPad Software Inc., La Jolla, CA)

### Reagents and consumables

• Custom peptides (> 95% pure) can be obtained from a number of commercial sources. In our hands, peptides from Selleck Chemicals (Houston, TX) have worked well.
• S100B, purified from bovine brain (Cat. # 559290; Merck Millipore, Darmstadt, Germany)
• Sensor Chip CM5 (single: Cat. # BR-1003-99; pack of three chips: Cat. # BR-1000-12; GE Healthcare)
• Plastic Vials, Ø 7 mm; pack of 1000 vials (Cat. # BR-1002-12; GE Healthcare)
• Rubber Caps, type 3; pack of 600 caps (Cat. # BR-1005-02; GE Healthcare)
• Immobilization buffers: 10 mM sodium acetate, pH 4.0 (Cat. # BR-1003-49) and pH 4.5 (Cat. # BR-1003-50; GE Healthcare); 1 × 50 ml each
• HBS-EP (Cat. # BR100188, 6 x 200 ml; GE Healthcare)
• NaOH (Cat. # S8045; Sigma-Aldrich, Stockholm, Sweden)
• DMSO (Cat. # D2650; Sigma-Aldrich)
• Amine Coupling Kit (contains EDC, NHS and 1.0 M ethanolamine-HCl pH 8.5; Cat. # BR100050; GE Healthcare)
• HEPES (Cat. # H3375; Sigma-Aldrich)
• NaCl (Cat. # S7653; Sigma-Aldrich)
• CaCl_2_ · 2H_2_O (Cat. # C8106; Sigma-Aldrich)
• Tween-20 (Cat. # P1379; Sigma-Aldrich)
• EDTA disodium salt (Cat. # E5134; Sigma-Aldrich)
• Syringe filters, 0.2 µm pore size (Cat. # 83.1826.001; Sarstedt, Helsingborg, Sweden)
• Bottle top filtration unit, 0.45 µm pore size (Cat. # 83.1823.100; Sarstedt)

### Recipes

All “home-made” buffers and solutions must be filtered to remove particles. Depending on volume, filtration can be done using syringe filters or bottle top filtration units.

*Running buffer with Ca2+*: 10 mM HEPES, 150 mM NaCl, 4 mM CaCl_2_, 0.005% Tween-20; pH 7.5. Store at 4˚C between experiments. Pass through bottle top filtration unit at least once. Allow to come to room temperature, then degas before use.

*Regeneration solution*: 10 mM NaOH, 10 mM EDTA. Pass through syringe filter at least once and degas before use.

**Caution**: Extended use of running buffers containing high concentrations of Ca^2+^ can be problematic, as Ca^2+^ tends to precipitate in the SPR instrument over time. This becomes apparent as an increase in baseline responses and can ultimately damage the system. Therefore, when using buffers with high Ca^2+^ concentrations, it is important to flush the system either with Ca^2+^-free or even EDTA-containing buffer in between runs. It is also imperative to follow routine cleaning procedures, such as by running “desorb” and “sanitize” programs as prescribed by the instrument control software. Further, make sure that there is no salt buildup on the connector block.

**Figure 1.**
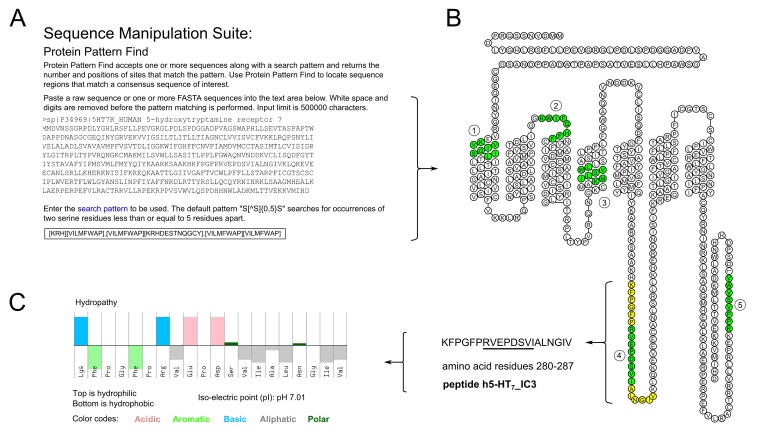
**Identification of putative S100B consensus motifs within the human 5-HT_7_receptor and selection of a peptide from the third intracellular loop. A.** Sequence analysis is used to identify putative consensus motifs. **B.** The motifs are mapped onto the topology of the target protein (see residues highlighted in green; spacers highlighted in yellow). Prior knowledge dictates which of the motifs are likely to be functionally relevant and guides the selection of peptides for further study. In the present case, five motifs exist overall but #4 (RVEPDSVI), located in the third intracellular loop (IC3) of the 5-HT_7_ protein, is prioritized based on prior knowledge of GPCR structure and function. **C.** Peptide property calculators are used to estimate physicochemical characteristics, such as isoelectric point and hydrophobicity of the peptide designed in this workflow.

## PROCEDURE

Routine preparation and maintenance of the Biacore 3000 instrument require the regular use of certain procedures (priming, flushing etc.) controlled by the “wizard” of the instrument software. Procedures thus not belonging to the molecular interaction assay *per se* are not listed here.

### Identification of putative binding motifs by sequence analysis

Putative binding sites for interacting proteins can be identified using bioinformatics. The only requirement for our present approach is the existence of a “consensus motif” that can be used as a search image in sequence analysis. Peptides derived from GPCR (e.g., 5-HT_1A_ and mGluR_7_) that contain a motif for binding of calmodulin have been successfully used in SPR studies [9, 11]. We recently discovered an S100B motif in the 5-HT_7_ receptor and used SPR to show that it mediates binding of S100B [15]. Our sequence analysis approach works as follows:

1. Retrieve the protein sequence representing your GPCR of interest. The official reference sequence provided by UniProt (www.uniprot.org) is a good starting point, unless experimental circumstances (interest in mutations, truncated variants etc.) dictate otherwise.
2. Navigate to the Sequence Manipulation Suite’s “Protein Pattern Find” module (www.bioinformatics.org/sms2/protein_pattern.html, [16]) and enter the protein sequence.
3. Define the search pattern. The S100B consensus motif as described by Ivanenkov and others [17] is +OXO*XOO, where amino acid residues are coded as follows: +, basic; O, hydrophobic; *, hydrophilic; X, variable. We translate this to [KRH][VILMFWAP].[VILMFWAP][KRHDESTNQGCY].[VILMFWAP][VILMFWAP], where letters in square brackets denote amino acids grouped according to properties (basic, hydrophobic, hydrophilic) and periods represent variable residues (**Fig. 1A**).
4. Retrieve the search results. If there are multiple hits, prioritize them according to biological criteria. Location within the target GPCR’s topology (e.g., intra- versus extracellular) will define whether a given hit is likely to be i) a “true positive” and ii) relevant to your experiment or not. Databases such as GPCRDB (http://tools.gpcr.org) can help in this task, as they predict extra- and intracellular domains as well as transmembrane portions and can visualize them in the form of “snake plots” (**Fig. 1B**).

### Design and optimization of receptor domain peptides

Based on the results from sequence analysis, receptor domain peptides can be designed that encompass the putative binding motif(s). Design criteria will vary from project to project, but the following notes should be considered:

5. Use peptide property calculators (see Appendix) to estimate the biochemical characteristics of your peptides (**Fig. 1C**). If properties are unfavorable (e.g., extreme isoelectric point or hydrophobicity), consider adding terminal residues that counteract these properties. Attention to this step will greatly support the entire experimental protocol. Right at the start, it will guide the choice of solvent (e.g. DMSO for hydrophobic peptides, aqueous solvents for hydrophilic peptides) and immobilization buffer for a given peptide.
6. Include “spacers” upstream and downstream of the motif. In the present example, the S100B motif (eight amino acids) is flanked by six amino acids each at its N- and C-terminus (see residues highlighted yellow in **Fig. 1B**) for a total of 20 amino acid residues. Thus, the sequence of peptide h5-HT_7__IC3 is KFPGFPRVEPDSVIALNGIV (S100B motif underlined).
7. Consider adding functional groups to your peptides, e.g., by ordering them in biotinylated form. This enables standardized coupling procedures (Sensor Chip SA; Cat. # BR100398, GE Healthcare), which in turn facilitates the generation of similar surface densities with defined ligand orientation. When series of closely related peptides need to be studied (wild-type, mutant, +/- posttranslational modification), this approach can be very useful.

### Preparation of stock solutions of peptides and interacting protein

Peptides vary greatly in their biochemical characteristics. Despite the utility of peptide property calculators (see Appendix), appropriate solvents thus need to be determined empirically and problems can occur (see **Table 1**, Troubleshooting). We proceeded as follows:

8. Peptide h5-HT_7__IC3 (hydrophobic): Allow the peptide source vial to come to room temperature before opening. If possible, add DMSO directly to the source vial for a final peptide concentration of 20 mg/ml. For example, add 100 µl DMSO to 2 mg of peptide. Dissolve the peptide by gently agitating the source vial, i.e. rolling it back and forth between the tips of your fingers. Transfer the peptide solution to a glass vial, tightly cap the vial and store at -20°C.
9. S100B: Add 200 µl of 50 mM Tris-HCl, pH 8.0 (passed through a syringe filter before use) directly to the source vial for a final concentration of 5 mg/ml (~ 240 µM). Dissolve S100B by gently agitating the vial or by pipetting up and down. Store as aliquots at -20°C.

### Preparation of serial dilutions of peptide (ligand) and S100B (analyte)

Stocks of peptides and proteins should be stored frozen. Dilutions should be made fresh on the day of use, as follows:

10. Peptide h5-HT_7__IC3: Add 1 µl of peptide stock solution (20 mg/ml) to 399 µl of 10 mM sodium acetate pH 4.0 or 4.5, yielding 50 µg/ml. Further dilute 1:2 with 10 mM sodium acetate pH 4.0 or 4.5, yielding 25 µg/ml. Final concentration of DMSO is 0.25% or 0.125%, respectively. Dispense diluted peptide into plastic vials (Ø 7 mm) and close with rubber caps.
11. S100B: Add 45 µl of stock solution (240 µM) to 225 µl running buffer, yielding 40 µM. Add 130 µl of 40 µM solution to 130 µl running buffer, yielding 20 µM. Serially dilute down to 1.25 µM. Alternatively, add 15 µl of stock to 285 µl of running buffer, yielding 12 µM. Add 130 µl of 12 µM solution to 130 µl running buffer, yielding 6 µM. Serially dilute down to 0.38 µM. For every concentration of S100B, dispense duplicates of 65 µl each in plastic vials and close with rubber caps. Include duplicate vials of running buffer as blanks.

### Pre-concentration assays (pH scouting)

**Figure 2.**
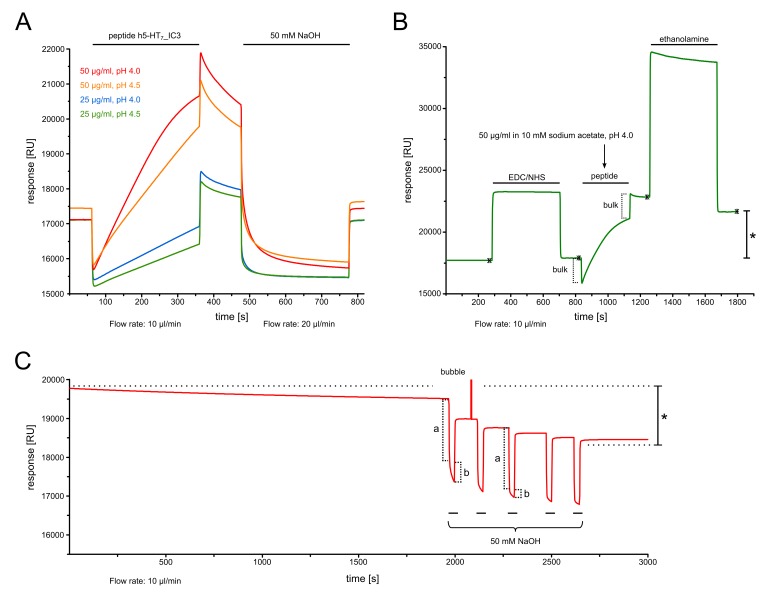
**Pre-concentration, peptide immobilization and conditioning of the newly generated sensor surface. A.** Peptide h5-HT_7__IC3 is dissolved in immobilization buffer (10 mM sodium acetate) at various concentrations and pH values. Injections (black horizontal bars) over the CM5 chip reveal concentration- and pH-dependent electrostatic attraction (“pre-concentration”) to the negatively charged carboxymethylated dextran matrix on the chip surface. Notably, a sizable fraction of pre-concentrated h5-HT_7__IC3 is retained at the surface, i.e. does not completely dissociate after injection (see period before injection of 50 mM NaOH). Also note the large bulk response, i.e. a sharp and sudden decrease in RU values upon peptide injection, which arises because the peptide is dissolved in DMSO. **B.** Immobilization of peptide (50 µg/ml in 10 mM sodium acetate, pH 4.0) occurs after activation of the chip surface with EDC/NHS. The surface is then deactivated with 1.0 M ethanolamine-HCl, pH 8.5. Note bulk response upon peptide injection, as expected from pre-concentration assays. Asterisk indicates the amount of peptide immobilized according to the Biacore software wizard. **C.** Conditioning of the sensor surface through injections of 50 mM NaOH (horizontal bars) and stabilization of baseline in running buffer overnight. Initially, the sensorgram slowly drifts towards lower RU values. Letters a and b indicate bulk responses to NaOH and removal of “pseudo-immobilized” peptide, respectively. Note how the extent of removal diminishes between injections 1-5. The distance between the two dotted horizontal lines (see asterisk) represents the difference between “apparent” (per the software wizard) and “true” amount of immobilized peptide. Towards the end of the observation period, the baseline is stable and horizontal, showing that the surface is now ready for use.

12. To determine the optimal pH for immobilization, prepare the peptide ligand in various immobilization buffers (see step 10) for “pH scouting” and place corresponding plastic vials in the Biacore 3000’s autosampler.
13. In the instrument control software, go to “File → New Application Wizard → Surface Preparation → Immobilization pH Scouting” and specify flow rate, injection volume etc.
14. Monitor pre-concentration of the peptide (electrostatic attraction to the sensor chip surface) in real-time and look for bulk effects (changes in response due to difference in refractive index of solutions), speed and magnitude of pre-concentration, dissociation after each injection ends etc. To minimize bulk contributions from the peptide solvent, prepare stocks in DMSO at the highest possible concentration of peptide (i.e. lowest possible concentration of DMSO in the assay).
**Note**: In **Figure 2A**, pre-concentration assays for peptide h5-HT_7__IC3 are shown. Two concentrations (25 or 50 µg/ml) were prepared in 10 mM sodium acetate (pH 4.0 or 4.5). Injection volume was 50 µl. Flow rate of the running buffer (HBS-EP) was 10 µl/min. Notice the large negative change in response (bulk effect) upon peptide injection, which is mainly caused by the peptide solvent (DMSO). Also note post-injection retention (i.e. incomplete dissociation) of pre-concentrated material, most likely due to the peptide’s hydrophobicity (**Fig. 2A**).

### Immobilization

15. Based on results from pH scouting (see above), prepare peptide h5-HT_7__IC3 for immobilization in 10 mM sodium acetate pH 4.0 to a final concentration of 50 µg/ml.
16. Start an immobilization program via the Biacore 3000’s software wizard (“File → New Application Wizard → Surface Preparation → Immobilization”) and select amine coupling with a CM5 chip.
17. Select the flow cell (e.g., Fc4) in which the peptide will be immobilized.
18. Set flow rate to 10 µl/min and injection time to 5 min.
19. Place plastic vials with EDC (115 µl), NHS (115 µl), peptide h5-HT_7__IC3 (90 µl) and ethanolamine (75 µl) in the instrument’s autosampler together with one empty plastic vial for mixing of EDC and NHS.
20. Execute the program and carefully inspect the results, using both the numbers returned by the software wizard and (**important!**) the raw sensorgram.
**Note**: As shown in **Fig. 2B**, the complete immobilization procedure entails activation of the surface via injection of mixed EDC/NHS, injection of peptide h5-HT_7__IC3 and finally deactivation of the surface with ethanolamine. According to the software wizard, the amount of peptide bound (“response final”) was approximately 4000 RU. However, given the degree of post-injection retention observed during pH scouting (see above), we decided to “condition” the newly generate sensor surface in order to reveal the amount of peptide that was actually covalently immobilized (see following section).
21. Repeat the immobilization procedure (steps 16-20) in a different flow cell (e.g., Fc3), using running buffer instead of diluted peptide. This will create a “mock-immobilized” reference surface.
**Note**: In a given SPR experiment, unspecific binding responses can be subtracted from true responses by using the Biacore 3000’s capability for in-line reference subtraction. This requires preparation of a reference surface in addition to the actual sensor surface containing the ligand of interest. Several options exist for the preparation of such a reference surface, and there is no “one-size-fits-all” solution. Commonly used are unmodified surfaces (the simplest approach) and surfaces holding “inert” ligands (e.g., proteins that are known not to interact with the analyte). In the present protocol, a “mock-immobilized” surface is prepared by running the immobilization procedure described above in the absence of peptide h5-HT_7__IC3. Thus, the surface is activated with EDC/NHS, then presented with running buffer, and finally deactivated with ethanolamine.

For a thorough discussion of immobilization strategies via amine coupling, see [18].

### Conditioning of newly generated sensor surfaces

22. Start a sensorgram (“Run → Run Sensorgram”) and observe the baseline response over an extended amount of time. Check whether the baseline is fairly stable when only running buffer is flowing through the system.
23. If the baseline appears to be drifting downward, inject a short pulse of 50 mM NaOH. Continue to observe the baseline.
24. Inject additional pulses of 50 mM NaOH as needed and allow the baseline to stabilize. Leave the sensorgram running overnight, with running buffer flowing through the system.
25. Check and make sure that the baseline is stable (i.e. the sensorgram proceeds horizontally rather than drifting downward or upward) before using the new sensor surface.
**Note**: In **Figure 2C**, one can see a slow and steady decrease in the baseline response of a newly generated sensor surface, indicating that some of the pre-concentrated peptide was not actually covalently coupled. Repeated injections of 50 mM NaOH were therefore used to remove “pseudo-immobilized” peptide (most likely retained through hydrophobic interactions with the CM5 chip’s surface). Towards the end of the observation period shown in **Fig. 2C**, the baseline response was no longer decreasing. After further stabilization in running buffer overnight, the actual amount of peptide immobilized was estimated to be 1200 RU, i.e. sizably lower than the 4000 RU indicated by the software wizard. This illustrates that all sensorgrams have to be carefully analyzed in order to determine the “true” immobilization level. Notably, an independent immobilization of peptide h5-HT_7__IC3 yielded almost identical results (data not shown), the only difference being that 100 mM HCl alternating with 200 mM NaOH was used for conditioning.

### Real-time measurement of molecular interactions

26. Place the vials containing serial dilutions of S100B (see step 11) in the instrument’s autosampler, together with vials containing regeneration solution.
27. Prime the system three times with running buffer before each experiment.
28. In the instrument control software, specify the flow cell that contains the sensor surface and the flow cell for in-line reference subtraction. For the procedure used in the present protocol, detection was set to “Fc4-3” (sensor surface in Fc4, reference surface in Fc3).
29. Specify injection of each sample (serial dilution of S100B) as a volume of 25 µl at a flow rate of 5 µl/min.
30. Specify injection of regeneration solution (between cycles of sample injection) as a volume of 10 µl at a flow rate of 10 µl/min.
31. Set temperature control to 25˚C and execute the program. At the end of the experiment, all data will be saved as a “Biacore result file”.
**Note**: When a given sensor chip is used repeatedly, it is important to monitor stability of the baseline response over the course of several subsequent interaction analyses. If the baseline does not change much, one can assume that immobilization and conditioning of the sensor surface worked well and corresponding measurements should be reliable.

### Primary data analysis – Deriving steady-state responses from sensorgrams

In cases where the on/off rates for a given interaction are too fast to be resolved by the SPR instrument (see [11] for an illustrative example), measurements of affinity (Kd) are derived via steady-state rather than kinetic methods.

**Figure 3.**
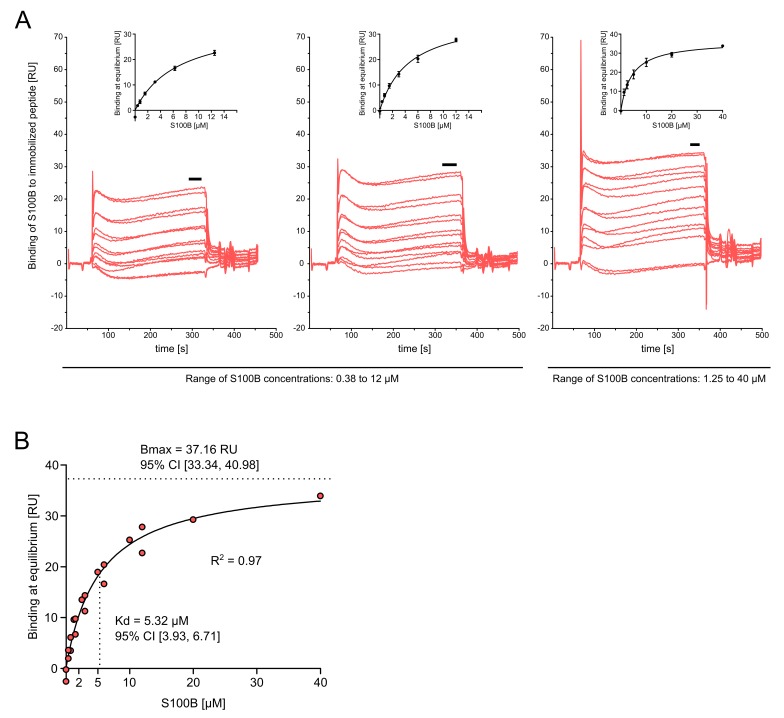
**Sensorgrams reveal binding of S100B to peptide h5-HT_7__IC3 and allow derivation of affinity estimates through equilibrium analysis and nonlinear regression. A.** Three experiments were carried out with a CM5 chip to which h5-HT_7__IC3 had been immobilized. Concentrations of S100B (in duplicate) ranged from 0.38 to 12 µM in the first two experiments (left and middle panel). Because curve fitting via nonlinear regression (see insets) indicated that saturation had not been reached, the concentration of S100B was increased from 1.25 to 40 µM in the third experiment (right panel). Note that the fitted curve now more closely approaches saturation. Black horizontal bars indicate those parts of the sensorgrams from which steady-state data (binding response at equilibrium, in response units [RU]) were derived. **B.** Data from the three experiments were summarized by plotting the binding responses at equilibrium against the concentrations of S100B and fitting a one-site binding model. Each point represents averaged duplicates from a given experiment. The affinity of interaction (dissociation constant, Kd) and maximal binding capacity of the sensor surface (Bmax) were derived and are listed with 95% confidence intervals (CI). The R^2^ value indicates goodness of fit for the one-site binding model.

32. Open your result file using the BIAevaluation software. Inspect all sensorgrams carefully, cycle by cycle, to check for anomalous responses (e.g., caused by air bubbles) and other conspicuous features. Exclude cycles from subsequent analysis if necessary.
33. Select those sensorgrams that contain reference-subtracted responses (from Fc4-3 in our case) and click the “Plot Overlay” button in the software.
34. Cut off those parts of the sensorgrams that contain large response artefacts towards the end of each cycle. Highlight the baseline at the beginning of each cycle and set it to zero by clicking “Calculate → Y-Transform → Zero at Average of Selection”.
35. Highlight an area of the sensorgrams at which analyte binding has reached equilibrium, typically towards the end of the analyte injection (see **Fig. 3A**). Click “Fit → General → Average” and enter the analyte concentrations that correspond to a given injection cycle in the field labeled “Conc”.
**Note**: To perform steady-state analysis as described above, the model “Average” first has to be imported into the BIAevaluation software. Click “File → Import Models” and navigate to folder “BIAeval” (usually under C:\Program Files or C:\Program Files (x86)). Click file “More Models.mdl” and import “Average” plus any other models you might need.
36. Click “Fit” and then navigate to the “Parameters” tab in the lower left corner of the screen. Copy data in columns “Req” (equilibrium response) and “Conc” and proceed to step 37.
**Note**: At this stage, it is important to confirm that the Req values derived from fitting are reasonable. Their corresponding Chi^2^ value (see tab “Report”) should be small and the residuals (see tab “Residuals”) should scatter around zero by no more than 10% of the measured response. See also http://www.sprpages.nl/data-fitting/validation.html for additional useful information.

### Secondary data analysis – Non-linear regression of steady-state data

37. The steady-state binding responses derived in steps 32-36 are used to determine the affinity between ligand (peptide h5-HT_7__IC3) and analyte (S100B). For this purpose, transfer the corresponding data into GraphPad Prism and plot Conc (X) against Req (Y) in an XY table. Plot replicate values of Req (e.g., from duplicate determinations) separately.
38. Fit a one-site binding model to the data by choosing “Analyze → Nonlinear regression (curve fit) → Binding - Saturation → One site - Specific binding”. The results sheet will list parameters “Bmax” (maximum binding capacity of the sensor surface; “Rmax” in Biacore terminology) and “Kd” (dissociation constant, affinity) together with their 95% confidence intervals (CI). Value R^2^ (“R square”) is listed as an indicator of the “goodness of fit”.
39. Carefully inspect all parameter estimates derived via curve fitting. The 95% CIs should be narrow and the R^2^ should be close to 1.
**Note**: The interaction analysis described above should be repeated several times with a given sensor surface, using replicates for each analyte concentration in each experiment. Affinity estimates can be derived from each individual experiment and then summarized into one value (with 95% CIs and ± error, if desired). Once an experimental series is concluded, it makes sense to plot all data in one summary file. Consider showing a scatter plot (see **Fig. 3B**) to illustrate how individual data points are distributed relative to the curve defined by nonlinear regression. To further corroborate experimental findings, an independent sensor surface should be generated and the interaction analysis repeated. The observed affinity between ligand and analyte should be very similar across experiments and sensor surfaces.

## ANTICIPATED RESULTS

Figures 1 through 3 show results for all major steps of the procedure, including ligand pre-concentration, immobilization, conditioning of the surface, real-time sensorgrams of analyte binding and nonlinear regression analysis of steady-state response data. Typical features of our results are as follows (see also [15]):

*Pre-concentration*: Depending on their concentration and the pH of immobilization buffers, peptides will pre-concentrate at the surface of the CM5 chip via electrostatic attraction. Responses are typically well over 1000 RU. Hydrophobic peptides can show post-injection retention, i.e. might not completely dissociate from the surface (**Fig. 2A**).

*Immobilization*: Using peptides such as h5-HT_7__IC3, final amounts immobilized on a CM5 chip via amine coupling have been around 1200 RU in our hands. This is similar to what Turner and others have achieved (400-720 RU) with IC3 domain peptides from the 5-HT_1A_ receptor [11] and practically identical to the “immobilization threshold of 1000 RU” suggested by Leclerc for interaction analyses involving RAGE domain peptides and S100 proteins [19]. Higher amounts are not necessarily desirable, as mass transfer effects eventually occur. Note that “final immobilized amounts” were revealed only after conditioning of the new sensor surface (**Fig. 2C**), the requirements for which will vary with each peptide of interest.

*Real-time measurement of molecular interactions and data analysis*: Our results (**Fig. 3**) are very similar to published studies in which interactions between immobilized peptides and S100B were analyzed by SPR-based approaches (see e.g., [20-22]). The study by Ostendorp and others indeed shows almost identical sensorgram shapes and affinity of interaction, as well as similar response magnitudes (see **Fig. 6A** in [22]). Using IC3 domain peptides from the 5-HT_1A_ receptor (a family member of 5-HT_7_) and calmodulin (a family member of S100B), Turner and others found sensorgram shapes (very fast on/off rate), response magnitudes (< 100 RU) and apparent affinities that are very similar to our results with peptide h5-HT_7__IC3 and S100B [11]. The same is true for C-terminal domain peptides from various GPCR and their interactions with PSD95 [10]. Interactions between calmodulin and C-terminal peptides from mGluR7 also show very fast on/off rates [9], suggesting that this is a common feature of interactions between GPCR and calcium-binding proteins. Our “typical results” shown here are thus in excellent agreement with the literature and highlight the robustness of the method described in the present protocol. This notion is further supported by non-SPR data from interactions between S100B and IC3 of the dopamine D_2_ receptor, the apparent affinity of which is almost identical to our results [23].

## TROUBLESHOOTING

**Table 1.** Troubleshooting.

Step	Problem	Cause	Suggestions
8	Peptide insoluble	Extreme physicochemical properties, such as hydrophobicity	Consider adding a few terminal residues that favor solubility
14	Poor peptide pre-concentration; large bulk effects	Extreme physicochemical properties, such as isoelectric point (pI)	Consider adding a few terminal residues that alter the pI; start from high peptide stock concentrations to minimize DMSO bulk effects
28-31	No interaction apparent in the assay	Running buffer inappropriate (*e.g.*, in terms of ion concentrations); insufficient density of ligand at the sensor surface and/or insufficient analyte concentration	Optimize the running buffer by systematically altering its components; aim for higher level of ligand immobilization and use sufficiently high concentrations of analyte

### Advantages of the method

Receptor domains are readily available in the form of custom-synthesized peptides. Flexibility in designing these peptides is a key advantage of the present approach. For instance, when sequence analyses indicate the presence of putative phosphorylation sites or naturally occurring mutations in a given receptor domain, these features can immediately be incorporated into custom peptides. The influence of these features on the affinity and kinetics of S100B binding can then be studied (see also “SPR analysis of protein fragments and mutants”; [7]), enabling rapid testing and refinement of experimental hypotheses. If a given feature (e.g., phosphorylation site) indeed affects binding, corresponding receptor mutants can be generated and interrogated in live cells. The speed with which a putative binding site can thus be confirmed or falsified is a clear advantage of using the present SPR approach.

### Drawbacks and limitations

One limitation is that the interacting protein should be available in purified form. While many proteins are commercially available, some of the more “esoteric” ones can be costly and others simply are not on the market. In such cases, custom production and purification of recombinant proteins is necessary, which can be done either commercially or via collaborations. Nonetheless, because protein consumption is usually low, the present SPR method will be useful for many interacting proteins. Perhaps the main drawback stems from the fact that receptor domains rather than full-length receptor proteins are used. Results may therefore not always recapitulate physiological conditions, which can be mimicked in more sophisticated assay systems [13].

## Abbreviations used

5-HT_7_: serotonin 5-HT_7_ receptor
DMSO: dimethyl sulfoxide
EDC: 1-ethyl-3-(3-dimethylaminopropyl)carbodiimide hydrochloride
GPCR: G protein-coupled receptor
HBS-EP: HEPES-buffered saline with EDTA and surfactant P20
HCl: hydrochloric acid
IC3: third intracellular loop
NaOH: sodium hydroxide
SPR: surface plasmon resonance
NHS: N-hydroxysuccinimide
RU: response units.

## Peptide property calculators:

• http://protcalc.sourceforge.net/cgi-bin/protcalc
• https://www.genscript.com/ssl-bin/site2/peptide_calculation.cgi
• http://www.innovagen.se/custom-peptide-synthesis/peptide-property-calculator/peptide-property-calculator.asp

## Useful web resource for various SPR-related topics:

• http://www.sprpages.nl

## References

[1] Overington J. P., Al-Lazikani B., Hopkins A. L. (2006). How many drug targets are there?. Nat Rev Drug Discov.

[2] Hopkins A. L., Groom C. R. (2002). The druggable genome. Nat Rev Drug Discov.

[3] Katritch V., Cherezov V., Stevens R. C. (2011). Diversity and modularity of G protein-coupled receptor structures. Trends Pharmacol Sci.

[4] Lagerström M. C., Schiöth H. B. (2008). Structural diversity of G protein-coupled receptors and significance for drug discovery. Nat Rev Drug Discov.

[5] Adamson R. J., Watts A. (2014). Kinetics of the early events of GPCR signalling. FEBS Lett.

[6] Northup J. (2004). Measuring rhodopsin-G-protein interactions by surface plasmon resonance. Methods Mol Biol.

[7] Slepak V. Z. (2000). Application of surface plasmon resonance for analysis of proteinprotein interactions in the G protein-mediated signal transduction pathway. J Mol Recognit.

[8] Homola J. (2008). Surface plasmon resonance sensors for detection of chemical and biological species. Chem Rev.

[9] Isozumi N., Iida Y., Nakatomi A., Nemoto N., Yazawa M. (2011). Conformation of the calmodulin-binding domain of metabotropic glutamate receptor subtype 7 and its interaction with calmodulin. J Biochem.

[10] Møller T. C., Wirth V. F., Roberts N. I., Bender J., Bach A. (2013). PDZ domainmediated interactions of G protein-coupled receptors with postsynaptic density protein 95: quantitative characterization of interactions. PLoS One.

[11] Turner J. H., Gelasco A. K., Raymond J. R. (2004). Calmodulin interacts with the third intracellular loop of the serotonin 5-hydroxytryptamine1A receptor at two distinct sites: putative role in receptor phosphorylation by protein kinase C. J Biol Chem.

[12] Locatelli-Hoops S., Yeliseev A. A., Gawrisch K., Gorshkova I. (2013). Surface plasmon resonance applied to G protein-coupled receptors. Biomed Spectrosc Imaging.

[13] Navratilova I., Dioszegi M., Myszka D. G. (2006). Analyzing ligand and small molecule binding activity of solubilized GPCRs using biosensor technology. Anal Biochem.

[14] Stenlund P., Babcock G. J., Sodroski J., Myszka D. G. (2003). Capture and reconstitution of G protein-coupled receptors on a biosensor surface. Anal Biochem.

[15] Stroth N., Svenningsson P. (2015). S100B interacts with the serotonin 5-HT7 receptor to regulate a depressive-like behavior. Eur Neuropsychopharmacol.

[16] Stothard P. (2000). The sequence manipulation suite: JavaScript programs for analyzing and formatting protein and DNA sequences. Biotechniques.

[17] Ivanenkov V. V., Jamieson Jr G. A., Gruenstein E., Dimlich R. V. (1995). Characterization of S-100b binding epitopes. Identification of a novel target, the actin capping protein, CapZ. J Biol Chem.

[18] Fischer M. J. E. (2010). Amine coupling through EDC/NHS: a practical approach. Methods Mol Biol.

[19] Leclerc E. (2013). Measuring binding of S100 proteins to RAGE by surface plasmon resonance. Methods Mol Biol.

[20] Delphin C., Ronjat M., Deloulme J. C., Garin G., Debussche L. (1999). Calcium-dependent interaction of S100B with the C-terminal domain of the tumor suppressor p53. J Biol Chem.

[21] Duda T., Koch K., Venkataraman V., Lange C., Beyermann M. (2002). Ca(2+) sensor S100beta-modulated sites of membrane guanylate cyclase in the photoreceptor-bipolar synapse. EMBO J.

[22] Ostendorp T., Leclerc E., Galichet A., Koch M., Demling N. (2007). Structural and functional insights into RAGE activation by multimeric S100B. EMBO J.

[23] Dempsey B. R., Shaw G. S. (2011). Identification of calcium-independent and calcium-enhanced binding between S100B and the dopamine D2 receptor. Biochemistry.

